# Aggregated postpartum cerebral autoregulatory curves in normotensive individuals, preeclampsia with severe features, and superimposed preeclampsia with severe features

**DOI:** 10.1088/1361-6579/adfeb4

**Published:** 2025-09-17

**Authors:** Helen Woolcock, Maria Katsidoniotaki, Leonidas Taliadouros Meng, Noora Haghighi, Anne-Sophie van Wingerden, Aymen Alian, Whitney A Booker, Natalie A Bello, Randolph S Marshall, Ioannis A Kougioumtzoglou, Nils H Petersen, Eliza C Miller

**Affiliations:** 1 Department of Neurology, Columbia University, New York, NY, United States of America; 2 Department of Obstetrics and Gynecology, New York University, New York, NY, United States of America; 3 Department of Civil Engineering & Engineering Mechanics, Columbia University, New York, NY, United States of America; 4 Department of Anesthesiology, Yale University, New Haven, CT, United States of America; 5 Department of Obstetrics and Gynecology, Columbia University, New York, NY, United States of America; 6 Department of Cardiology, Cedars-Sinai Medical Center, Los Angeles, CA, United States of America; 7 Department of Neurology, Yale University, New Haven, CT, United States of America; 8 Department of Neurology, University of Pittsburgh, Pittsburgh, PA, United States of America

**Keywords:** cerebral autoregulation, preeclampsia, stroke, chronic hypertension, antihypertensive, postpartum

## Abstract

*Objectives*. Impaired cerebral autoregulation could contribute to postpartum stroke risk in individuals with preeclampsia. We modeled aggregated static autoregulatory curves in the postpartum period in individuals with no hypertension, preeclampsia, and chronic hypertension with superimposed preeclampsia. *Approach*. This is a retrospective analysis of data from a prospective observational study of postpartum participants. We measured continuous mean arterial pressure (MAP) with finger plethysmography and cerebral blood velocity (CBv) with transcranial Doppler within 2 weeks after delivery. Data were aggregated and group curves generated from normalized MAP and CBv data using 3rd order polynomial equations. We compared overall polynomial curve shapes between groups as well as pair-wise comparisons of autoregulatory range. *Main results*. A total of 73 participants were enrolled: 21 (29%) normotensive, 31 (42%) with preeclampsia and 21 (29%) with superimposed preeclampsia. Polynomial *S*-curves suggested a flatter plateau in the normotensive group compared with both preeclampsia groups, but the differences were not statistically significant. Autoregulatory range were wider in both preeclampsia groups than in the normotensive group, with a MAP range of 27.5 mmHg in the normotensive group, 43.2 mmHg in the preeclampsia group, and 31.5 mmHg in the superimposed preeclampsia group, but only the difference between the preeclampsia and normotensive groups reached statistical significance (*p* = 0.02). *Significance*. Static autoregulation curves generated using third-order polynomials showed distinct characteristics in postpartum participants with normotension, preeclampsia, and superimposed preeclampsia, and suggested a wider cerebral autoregulatory range in those with preeclampsia.

**Abbreviations**
CBF: Cerebral blood flow
TCD: Transcranial Doppler
SBP: Systolic blood pressure
DBP: Diastolic blood pressure
CBv: Cerebral blood velocity
MAP: Mean arterial pressure
Mx: Pearson correlation coefficient index using mean values
PetCO2: Partial pressure of end-tidal carbon dioxide
PaCO2: Arterial partial pressure of carbon dioxide

## 1. Introduction

Preeclampsia is a pregnancy-induced hypertensive disorder characterized by multi-organ vasculopathy and endothelial dysfunction (American College of Obstetricians and Gynecologists 2020). Preeclampsia is associated with increased maternal stroke risk, contributing to high levels of maternal morbidity and mortality globally (Khan *et al* 2006, Kuklina *et al* 2009, Elgendy *et al* 2021). The highest risk periods for maternal stroke are the peripartum and postpartum periods, up to 6–12 weeks postpartum (Ban *et al* 2017, Meeks *et al* 2020, Elgendy *et al* 2021). Studies suggest that cerebral autoregulation, the brain’s homeostatic mechanism that minimizes variations in CBF when blood pressure is altered, may be impaired in patients with preeclampsia, possibly increasing the risk of neurovascular injury (van Veen *et al* 2013, Janzarik *et al* 2014, Van Veen *et al* 2015a, Janzarik *et al* 2019, Bergman *et al* 2021, Miller *et al* 2023).

Cerebral autoregulation is experimentally categorized and analyzed as static or dynamic (Zhang *et al* 1998, Tzeng and Ainslie 2014). Static cerebral autoregulation describes the steady-state relationship, over the course of minutes to hours, between MAP and CBF and reflects ultra-low blood pressure frequency oscillations (0–0.02 Hz or >50 s to complete one oscillation) (Brassard *et al* 2021, 2023). Conversely, dynamic cerebral autoregulation describes the spontaneous response of CBF to transient (over the course of seconds) changes in MAP and reflects very low (0.02–0.07 Hz or 50.0–14.3 s to complete one oscillation) to high frequency oscillations (0.20–0.35 Hz or 5.0–2.9 s to complete one oscillation) (Claassen *et al* 2016, Brassard *et al* 2021, 2023, Panerai *et al* 2023a, 2023a). Static cerebral autoregulation may be represented by a triphasic curve where, in healthy individuals, a longer and flatter plateau suggests a wider range of MAP where autoregulation is intact. This curve was first produced by Dr Niels Lassen by plotting the averaged or adjusted CBF and mean MAP in 376 participants across seven different studies, representing between-subject variations in the CBF–MAP relationship (Lassen 1959). Fluctuations in MAP were induced by vasoactive drugs in healthy individuals, and by including participants with pathological hypertensive states such as preeclampsia and chronic hypertension (Lassen 1959). Since the production of Lassen’s curve, several investigators have produced static autoregulatory curves by aggregating CBF and induced BP data from multiple studies, measured by TCD and other modalities (magnetic resonance imaging, Kety Schmidt technique, positron emission tomography, and radioisotope uptake studies) over the course of >2 min data collection periods (Numan *et al* 2014, Brassard *et al* 2021).

While Lassen included participants with preeclampsia in the original description of the static autoregulatory curve, no one has produced static autoregulatory curves using only data from participants with preeclampsia either in the antepartum or postpartum period. Here, we apply mathematical methods to successfully produce static autoregulatory curves using aggregated (group-level) data from a brief monitoring period. We illustrate characteristics of curves in postpartum participants with normotension, preeclampsia, and chronic hypertension with superimposed preeclampsia (‘superimposed preeclampsia’).

## 2. Materials and methods

### 2.1. Study design, population, and setting

**Figure 1.**
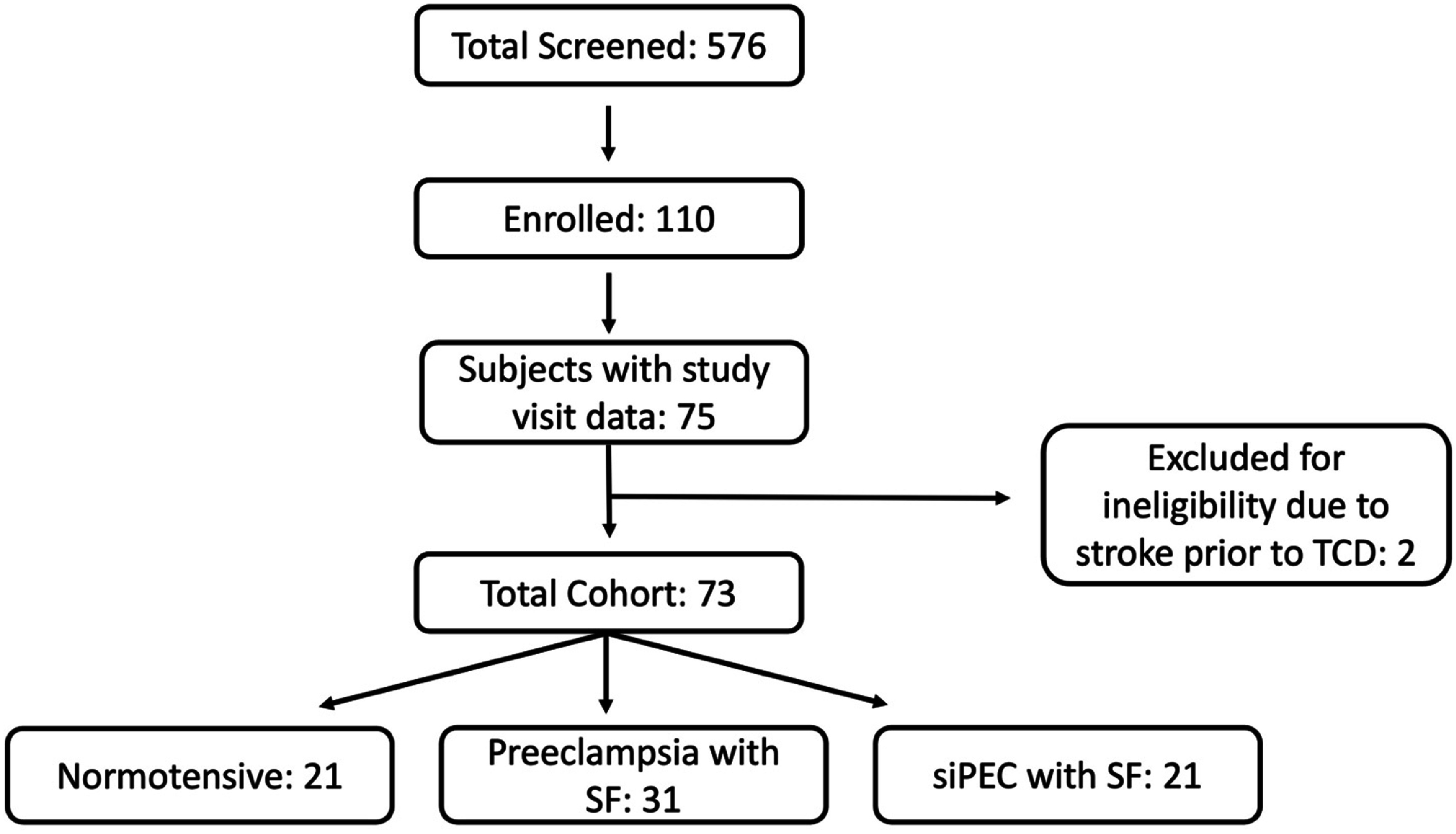
Flow diagram of MotherHealth study sample selection. Participants were screened in the antenatal or postpartum periods when they presented for care at the labor and delivery floor or antepartum floor between 28 September 2018, and 10 January 2023. Participants were approached for study enrollment if they met inclusion and exclusion criteria: participants with no prior stroke diagnosed with preeclampsia with severe features or normal blood pressure (no prior history of hypertension). Participants received CA studies in the early postpartum period, within 2 weeks after delivery. TCD = Transcranial Doppler. siPEC = chronic hypertension with superimposed preeclampsia.

This is a secondary retrospective analysis of data from the MotherHealth Study, a prospective observational cohort study of pregnant and postpartum individuals with and without preeclampsia with severe features, recruited at Columbia University Irving Medical Center (CUIMC) in New York City. Details of the study protocol and data collection methods have been previously described (Miller *et al* 2021). Preeclampsia with severe features was defined as SBP ⩾160 mmHg or DBP ⩾110 mmHg on two occasions at least 4 h apart after 20 weeks 0 d gestation, or new-onset signs of end-organ dysfunction as defined by the current practice guidelines of the American College of Obstetricians and Gynecologists (American College of Obstetricians and Gynecologists 2020). Participants with superimposed preeclampsia with severe features, defined as chronic hypertension with onset of preeclampsia with severe features after 20 weeks gestation, were also eligible for inclusion (American College of Obstetricians and Gynecologists 2019). The current analysis sample includes data collected from patients who presented to the labor and delivery floor or antepartum floor between 9/28/2018 and 1/10/2023 and received cerebral autoregulation studies at an early postpartum study visit, within 2 weeks of delivery. The 2 week threshold was chosen given the higher incidence of stroke within the first 2 weeks postpartum and the higher risk of hospital readmission for postpartum stroke within 10 d of discharge (Miller *et al* 2018, Too *et al* 2018, Sariyeva *et al* 2024). Participants with a history of ischemic stroke or intracerebral hemorrhage at any point prior to the autoregulation study were excluded (figure 1).

*Ethics statement:* The CUIMC Institutional Review Board approved all study procedures prior to beginning data collection (IRB-AAAR7300, approved 9 July 2018). The study was conducted in accordance with the principles embodied in the Declaration of Helsinki and in accordance with local statutory requirements. All participants provided written informed consent at the time of enrollment, prior to any procedures being performed.

### 2.2. Study measures

Participants lay supine while CBv and arterial blood pressure were collected simultaneously for 10 min following previously described procedures, as follows: (Skow *et al* 2022) left and right proximal middle cerebral arteries were insonated through the temporal window at a depth of 45–60 mm, using 2 MHz TCD probes mounted to a head frame (Claassen *et al* 2016, Skow *et al* 2022). After calibration, blood pressure was recorded via servo-controlled in-phase finger plethysmography (Finometer Pro, Amsterdam, Netherlands) (O’Brien *et al* 2001, Guelen *et al* 2003). Both the blood pressure and CBv signals were acquired at 100 Hz, a sampling rate which provides sufficient temporal resolution for both time-domain and frequency-domain analyses of cerebral autoregulation. Data were pre-processed with temporal synchronization of the blood pressure and CBv waveforms. Brachial blood pressure was measured once at each study visit with a validated arm-cuff device (Omron), using a standardized procedure (Miller *et al* 2021).

### 2.3. Data processing and mathematical modeling

*Generation of polynomial curves*. As there were no significant differences from side-to-side within individual participants, CBv from the left and right sides on the same person were averaged to generate the data for this analysis. Blood pressure readings via finger plethysmography and CBv were initially processed in two steps. Outliers were first removed by filtering extreme pressures (DBP >15 and <200, pulse pressure >15 and <250, and SBP >0 and <300) and removing the top and bottom 1% of the systolic and diastolic pressures. Due to the variability of baseline blood pressure between individuals, aggregated curves were produced across groups by normalizing MAP with a bandpass filter of (0.01, 0.1) Hz. The upper limit 0.1 Hz is equivalent to a 10 s moving average and the lower limit of 0.01 Hz removed the mean and any remaining shift due to measurement noise. The range of 0.01–0.1 Hz is widely accepted as the frequency range in blood pressure and CBv where cerebral autoregulation is best measured (Fantini *et al* 2016).

Cerebral autoregulatory curves were generated from normalized MAP and CBv data using 3rd order polynomial equations of the form *y = p*0 *+ p*1 * x + p2 * x^2^ + p3 * x^3^, to maximize goodness-of-fit. We used the Matlab function *p = polyfit*(*x,y,n*) that returns the coefficients for a polynomial *p*(*x*) of degree n (in this case predetermined as 3) that is a best fit (in a least-squares sense) for the data in *y*, where *x* is the pressure and *y* the flow velocity. Additional models or model selection criteria were not considered because of the simplicity of the polynomial model and given that the expected shape of the curves is known as *S*. Minimum and mean slope values at the plateau of the polynomial were calculated for each exposure group.

*Approximation of autoregulatory range*. To further illustrate autoregulatory range, we calculated the autoregulatory index Mx as a rolling Pearson correlation coefficient between 30 consecutive, 10 s averaged values of MAP and corresponding CBv values. This calculation is performed using a sliding window with 80% overlap, such that every 10 s, a new correlation is computed using the most recent 30 data points (i.e. a 5 min moving window), following established methods first described by Czosnyka and colleagues (Czosnyka *et al* 1997) and applied in prior autoregulation studies (Lee *et al* 2009, Zweifel *et al* 2010). Positive values of Mx indicate impaired autoregulation (i.e. a high positive correlation between CBv and MAP), whereas values near zero or negative indicate intact autoregulation (i.e. cerebral vasoreactivity effectively buffering changes in pressure) (Kostoglou *et al* 2024). These 30 data points were correlated, using a 2nd order polynomial, with normalized MAP to produce a U-shaped curve superimposed on the S-shape curve generated through the third order polynomials, with the nadir of the curve indicating the midpoint of the static autoregulatory curve. An accepted critical threshold of Mx = 0.3 was applied, such that Mx values >0.3 indicate higher CBF and MAP correlation and impaired autoregulation (Lang *et al* 2003, Petersen *et al* 2020). The width of the range between the upper and lower normalized MAP values at which the Mx crosses this 0.3 threshold was then estimated for each exposure group.

### 2.4. Statistical methods

Clinical and demographic characteristics were compared between exposure groups using *χ*^2^ for categorical variables and one-way ANOVA or Kruskal–Wallis for continuous variables, depending on the distribution. Autoregulatory curves were compared between the three primary exposure groups of interest: normotensive, preeclampsia, and superimposed preeclampsia. Since standard statistical comparisons between multiple groups of individuals were not applicable to curves generated from aggregated data, and reliable curves could not be generated for individual participants based on the short monitoring period, we took the following approach for statistical comparison of aggregate curves. For each participant, we fitted a 3rd order polynomial of the form *y = p*0 *+ p*1 * x + p2 * x^2^ + p3 * x^3^) to their processed MAP and CBV measurements, yielding four coefficients per subject. Given that some curves were based on very limited data, making them potentially unreliable, we considered these curves on individual participants as short samples from a distribution of the aggregated data forming part of the aggregate *S*-curve for each group. Treating each subject’s coefficient vector (*p*0*, p*1*, p*2*, p*3) as an independent sample within its clinical group, we applied a rank‐based multivariate Kruskal–Wallis test to compare the three groups’ four-dimensional coefficient distributions, followed by a 1000-replicate block-permutation to validate the *χ*^2^ approximations, which in small samples may be borderline accurate (Kruskal and Wallis 1952, Acar and Sun 2013). We also extracted the plateau width (horizontal distance between turning points) for each subject’s *S*‐curve and performed a univariate Kruskal–Wallis test on these one‐dimensional measurements, followed by a 1000-replicate permutation for validation (Elliott and Hynan 2011). Finally, we performed pairwise univariate Kruskal–Wallis tests for the one‐dimensional plateaus of the curves of the three groups (preeclampsia vs superimposed preeclampsia; preeclampsia vs controls; superimposed preeclampsia vs controls). An alpha of 0.05 was considered the threshold for statistical significance. We did not adjust for multiple comparisons given the exploratory nature of these analyses.

All analyses were performed using MATLAB [version 9.13 (R2022b), the MathWorks Inc, Natick, MA] and STATA/SE (version 16.1, StataCorp LLC, College Station, TX).

## 3. Results

### 3.1. Participant characteristics

A total of 73 participants contributed data to this analysis: 21 (29%) normotensive, 31 (42%) preeclampsia and 21 (29%) superimposed preeclampsia. Study visits were performed within fourteen days of delivery for all study participants. Participant characteristics are shown in table 1. Age and race or ethnicity were similar across groups whereas obesity (at time of delivery) was more common in participants with preeclampsia. Participants with preeclampsia delivered at an earlier gestational age than those with normotension. Among those with preeclampsia or superimposed preeclampsia, 23 (44%) had severe headache or visual disturbance at time of initial diagnosis. One participant experienced an eclamptic seizure more than 24 h prior to the study visit. Eight (11%) participants reported headache at the study visit (four in the preeclampsia group, three in the superimposed preeclampsia group, and one in the normotensive group). Brachial mean blood pressure measured at the study visit was higher in the preeclampsia and superimposed preeclampsia groups compared to the normotensive groups (*p* < 0.0001). Among all preeclampsia participants, 44 (85%) were treated with an antihypertensive within 24 h prior to the study visit, 10 (19%) were treated with intravenous magnesium sulfate within 24 h prior to the study visit, and 6 (12%) were treated with both antihypertensives and magnesium within 24 h prior to study visit.

**Table 1.** Characteristics of postpartum individuals with normotension, preeclampsia with severe features, and superimposed preeclampsia with severe features.

	Overall (*N* = 73)	Normotensive (*N* = 21)	PEC (*N* = 31)	siPEC (*N* = 21)	*p*-value
Characteristics					

Age at study visit, years (mean, SD)	33.4 (6)	34.8 (6)	32.1 (6)	34.1 (6)	0.26
BMI > 30 kg m^−2^ at delivery (*n*, %)	44 (60)	5 (24)	23 (74)	16 (75)	0.00
Primiparous (*n*, %)	39 (53)	12 (57)	16 (52)	11 (52)	0.92
Gestational age at delivery in weeks (median, IQR)	36 (34.1, 38.8)	39.29 (38.9, 39.6)	35.14 (33.3, 36.3)	34.3 (33.7, 36.3)	<0.0001
Gestational age at PEC diagnosis (median, IQR)	33.86 (30.9, 36.1)	—	34.71 (31.3, 36.3)	33 (30.6, 34.7)	0.17
Days from PEC diagnosis to TCD study (median, IQR)	3 (2, 5)	2 (2,3)	3 (3, 5)	5 (2, 24)	0.01
Days post-partum at study visit (median, IQR)	2 (2, 3)	2 (1, 2)	3 (2, 3)	2 (2, 4)	0.06
Preterm delivery (<37 weeks) (*n*, %)	43 (59)	1 (5)	26 (84)	16 (76)	<0.0001
Any prior adverse pregnancy outcome (*n*, %)	21 (29)	3 (14)	8 (26)	10 (48)	0.05
*HDP*	12 (16)	0	6 (19)	6 (29)	0.04
*Preterm*	11 (15)	1 (5)	3 (10)	7 (33)	0.02
*IUFD*	9 (12)	2 (10)	3 (10)	4 (19)	0.54

Physiological parameters and medications					

SBP in mmHg (mean, SD)	129.1 (17)	112.2 (11)	136.0 (14)	135 (15)	<0.0001
DBP in mmHg (mean, SD)	79.5 (10)	69.1 (8)	83.9 (7)	83.0 (9)	<0.0001
MAP in mmHg (mean, SD)	96.0 (12)	83.4 (8)	101.1 (8)	100.4 (11)	<0.0001
Continuous MAP in mmHg (mean, SD)	83.1 (17)	70.2 (14)	89.4 (13)	86.6 (16)	<0.0001
Antihypertensive 24 h from study visit (*n*, %)	44 (85)	—	25 (81)	19 (91)	0.34
*Only BB*	5 (10)	—	3 (10)	2 (10)	0.99
*Only CCB*	20 (38)	—	14 (45)	6 (29)	0.23
*Both BB and CCB*	8 (15)	—	3 (10)	5 (24)	0.17
*BB and CCB with ACEi or Diuretic*	2 (4)	—	0	2 (10)	0.08
Magnesium 24 h from study visit TCD (*n*, %)	10 (19)	—	5 (16)	5 (24)	0.49

*PEC = preeclampsia with severe features. siPEC = chronic hypertension with superimposed preeclampsia with severe features. BMI = body mass index. TCD = transcranial Doppler. HDP = hypertensive disorder of pregnancy. SBP = systolic blood pressure. DBP = diastolic blood pressure. MAP = mean arterial pressure. ACEi = angiotensin converting enzyme inhibitors. BB = beta blocker. CCB = calcium channel blocker.*

*SD = standard deviation. IQR = interquartile range. mmHg = millimeters of mercury. cm = centimeters. Kg = kilograms. N = number. % = percent.*

*Continuous MAP: measured by finger plethysmography. All other blood pressures measured by Omron arm cuff at study visit.*

*HDP defined as gestational hypertension or preeclampsia with or without severe features.*

### 3.2. Cerebral autoregulatory curves

**Figure 2.**
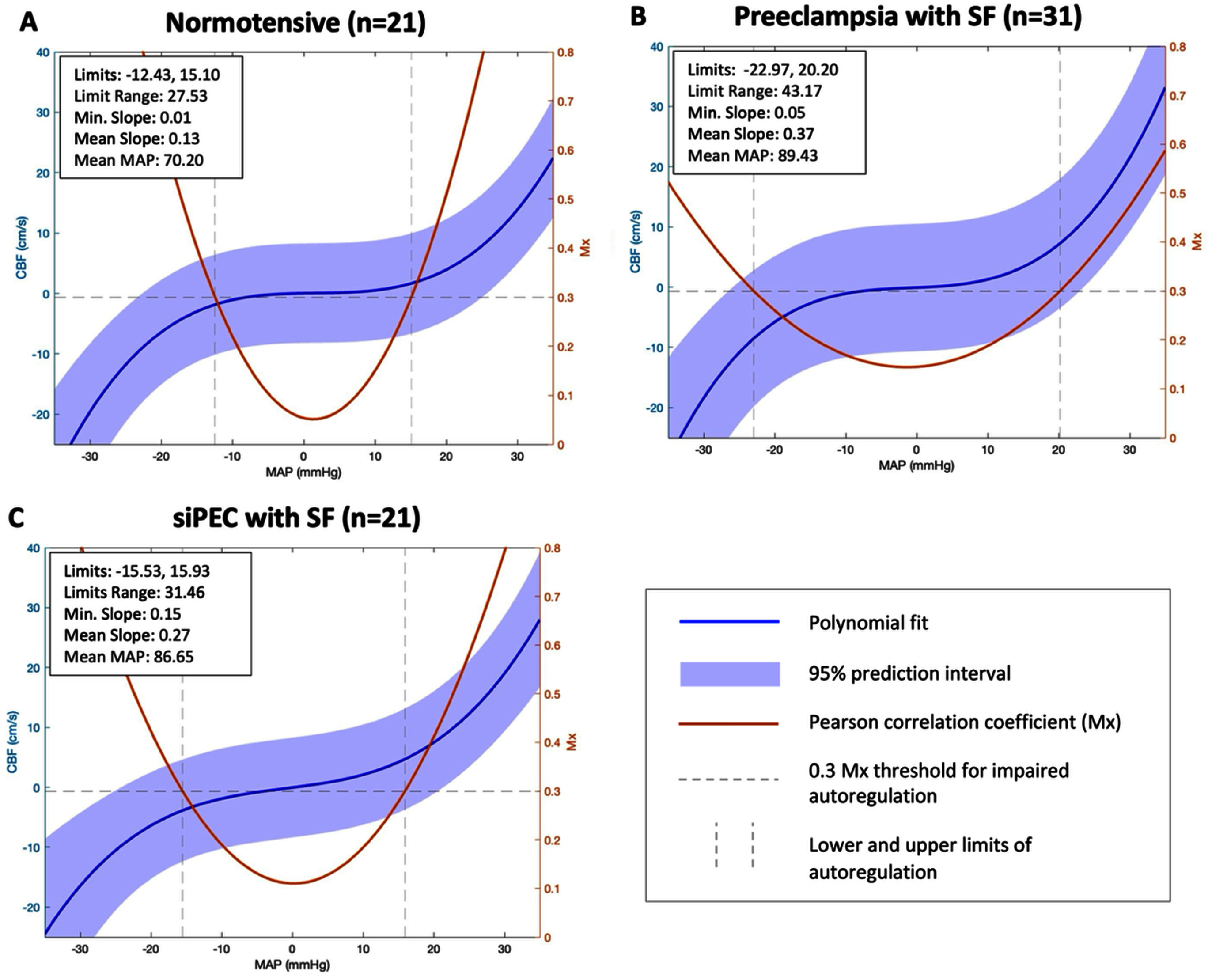
Static cerebral autoregulation in postpartum individuals with and without preeclampsia. Static cerebral autoregulatory curves were produced by aggregating data from early postpartum participants and comparing by exposure group: normotension, preeclampsia with severe features, or siPEC with severe features. The purple curve indicates the polynomial *S*-curve produced from aggregated blood pressure and CBF values. The orange curve indicates the *U*-curve of Mx, a moving Pearson correlation coefficient between CBv and MAP. An Mx approaching 0 indicates optimal cerebral autoregulatory function. The Mx critical threshold of 0.3 is applied (horizontal dotted lines), where Mx values >0.3 indicate impaired cerebral autoregulation. MAP is normalized to the mean. Upper and lower limits of autoregulation, denoted by the vertical grey dotted line, are the normalized MAP values that intersect at the Mx threshold of 0.3, and correspond to the upper and lower inflection points in the sCA curve. Minimum and mean slope at the plateau of the purple *S*-curve were calculated for each curve. siPEC = chronic hypertension with superimposed preeclampsia. CBF = cerebral blood flow. CBv = cerebral blood velocity. MAP = mean arterial pressure. Mx = Pearson correlation coefficient index. Min. = Minimum.

Polynomial *S*-curves demonstrated a minimum slope of 0.01 and mean slope of 0.13 at the plateau in the normotensive group (figure 2(A)). In the preeclampsia group, minimum slope was 0.05 and mean slope was 0.37 (figure 2(B)). In the superimposed preeclampsia group, minimum slope was 0.15 and mean slope was 0.27 (figure 2(C)). Coefficients of the best-fit polynomial equations for each group are shown in table 2. The asymptotic three-way *χ*^2^ comparison between the four-dimensional coefficient distributions resulted in *p* = 0.41, and a 1,000-replicate block-permutation confirmed this result with *p* = 0.42, indicating no significant group‐level differences in overall curve shape. Similarly, there were no significant group-level differences in autoregulatory range (*χ*^2^ approximation, *p* = 0.41; 1,000-replicate permutation, *p* = 0.40). Considered pairwise, autoregulatory range was wider in the preeclampsia group (MAP range 43.2 mmHg) compared to the normotensive group (MAP range 27.5 mmHg) (*p* = 0.02), and trended towards a difference between the superimposed preeclampsia group (MAP range 31.5 mmHg) vs the normotensive group (*p* = 0.07) (figure 2(C)). There was no significant difference in autoregulatory range between the preeclampsia group and the superimposed preeclampsia group.

**Table 2.** Third order polynomials describing aggregate autoregulatory curves in normotensive, preeclampsia, and superimposed preeclampsia groups.

Group	*n*	*p*3	*p*2	*p*1	*p*0
Normotensive	21	0.0006	−0.0032	0.0192	0.0399
Preeclampsia	31	0.0007	0.0018	0.0483	−0.0659
Superimposed preeclampsia	21	0.0005	0.0015	0.1511	−0.0332

Coefficients of cerebral autoregulatory curves generated from normalized MAP and CBv data from each exposure group, using 3rd order polynomial equations of the form *y = p*0 + *p*1 * x + *p2* * *x^2^* + *p*3 * x^3^ where *x* is the MAP and *y*, the CBv.

## 4. Discussion

In this descriptive study, we characterized cerebral autoregulation in postpartum participants with normotension, preeclampsia, and superimposed preeclampsia, using aggregated data from brief individual monitoring periods. We demonstrated that even with limited data, our model produced aggregate autoregulatory curves illustrating the quality and range of effective autoregulatory function across groups.

Limited data describe cerebral autoregulatory function during pregnancy, and most have focused on dynamic cerebral autoregulation. Prior studies have shown that in pregnancy, the upper and lower limits shift to widen the limits of cerebral autoregulation (Cipolla *et al* 2012, Chapman *et al* 2013), and dynamic cerebral autoregulatory function appears preserved or improved during healthy pregnancy (Janzarik *et al* 2014, van Veen *et al* 2016). By contrast, in preeclampsia, studies found that dynamic cerebral autoregulation was impaired compared with normotensive pregnant women (Zunker *et al* 2000, Oehm *et al* 2006, van Veen *et al* 2013, Van Veen *et al* 2015a, Bergman *et al* 2021). Participants with preeclampsia also exhibit increased CBv of the middle cerebral artery and increased cerebral perfusion pressure, even after treatment for elevated blood pressure (Zunker *et al* 1995, Riskin-Mashiah and Belfort 2005, Sonneveld *et al* 2014). Autoregulation appears most impaired among those with superimposed preeclampsia (Van Veen *et al* 2015a).

We observed wider autoregulatory range in both preeclampsia and superimposed preeclampsia groups compared to the normotensive group (although only the preeclampsia vs normotensive comparison reached statistical significance), suggesting a possible greater capacity to cope with large blood pressure fluctuations. While the plateaus of both preeclampsia groups were numerically higher compared to the normotensive group, suggesting the possibility of a more pressure-passive relationship between MAP and CBv within the limits of autoregulation, these differences did not reach statistical significance.

While data on cerebral autoregulation in the postpartum period are limited, prior work using transfer function analysis showed impaired dynamic cerebral autoregulation (reduced phase shift, indicating a slower autoregulatory response) at 10 d postpartum in preeclampsia patients (Janzarik *et al* 2019, Claassen *et al* 2021). Another study of postpartum dynamic cerebral autoregulation showed higher gain (decreased dampening effect) in postpartum individuals with and without preeclampsia, compared to nonpregnant healthy female participants (Miller *et al* 2023). Our results cannot confirm these findings, but they suggest a promising methodology which may yield important insights into group autoregulatory characteristics in this hitherto understudied population.

### 4.1. Clinical implications

Several analytical models are available for cerebral autoregulation quantification, but none have gained widespread use in clinical practice. While static cerebral autoregulation is represented by the classical triphasic curve, dynamic cerebral autoregulation can be quantified by multiple methods. These include frequency-domain analysis techniques which are applied to blood pressure and CBv oscillations, such as transfer function analysis, and time-domain analyses, which quantifies the correlation between CBF and blood pressure or cerebral perfusion pressure using a Pearson correlation coefficient (Brassard *et al* 2023). Despite efforts to improve and refine these methods, there is no agreed gold standard clinical tool for cerebral autoregulatory interpretation and results are difficult to compare between methods (Claassen *et al* 2016, Brassard *et al* 2023, Panerai *et al* 2023a). Our model incorporated elements of time-domain analysis techniques by producing an Mx correlation coefficient between CBF and blood pressure, superimposed upon a static curve to provide a framework for understanding cerebral autoregulation within this patient population.

Patients with preeclampsia have high blood pressure variability (Jieyu *et al* 2019, Wingerden *et al* 2024), and may experience rapid and wide blood pressure fluctuations, particularly when receiving standard treatments such as antihypertensive medications or intravenous magnesium sulfate. In the setting of extreme blood pressure lability, decreased efficacy of the autoregulatory response could result in hyper- or hypoperfusion of the cerebral parenchyma (Miller 2019, Brassard *et al* 2021). Brassard *et al* recommend the necessity for close CBF and blood pressure monitoring in patients with high risk of cerebral hypo- or hyperperfusion (Brassard *et al* 2021). Closer blood pressure monitoring, potentially with real-time assessment of cerebral autoregulation function, may be warranted in the early postpartum period in patients with preeclampsia.

### 4.2. Research implications

Production of accurate cerebral autoregulatory curves typically requires wide fluctuations in MAP (induced by highly vasoactive medications or systemic hypertensive states) and invasive monitoring techniques (Strandgaard *et al* 1973, Strandgaard 1976, Larsen *et al* 1994). This allows for sufficient data points to produce accurate *S*-curves for individuals. In contrast, 10 min of non-invasive monitoring is generally considered insufficient time to adequately generate a static cerebral autoregulatory curve and identify personalized limits of autoregulation. As expected, our data, collected over a 10 min period per participant, did not show sufficient MAP fluctuations within individuals to produce reliable individual curves. For this reason, we performed this analysis using aggregated data from groups of participants, by normalizing MAP and CBv around the mean due to inter-individual differences in MAP and CBv values. Our curves provide us with a qualitative view of autoregulatory function across groups (between-subject), rather than individual (within-subject) personalized limits of autoregulation. We currently lack the mathematical tools to generate an accurate, personalized autoregulatory curve using only brief monitoring periods. We hope that this study will spark additional research in mathematics and data science that will ultimately lead to achieving this long-term goal, which could greatly increase the use of autoregulatory measures as useful tools for real time clinical decision-making.

### 4.3. Limitations

Our study has limitations. As noted above, we were unable to produce individual curves for each participant given the lack of intra-individual large blood pressure fluctuations within the 10 min monitoring period. With a longer monitoring period per patient, it may be possible to produce individual curves. Due to the use of compiled and normalized data points from each exposure group, we were only able to compare aggregate curves using multivariate extensions of standard statistical tests. In addition, the small number of participants may have introduced Type 2 error.

*Technical limitations*. We did not include measurement of PetCO2 as part of our study. Elevated levels of PaCO2 cause vasodilation; thus, cerebral autoregulation analysis protocols recommend accounting for PetCO2 when evaluating autoregulation (Brassard *et al* 2023). A prior study of postpartum patients found no differences in PetCO2 between normotensive patients and preeclampsia patients, while PetCO2 was lower in both groups in the early postpartum period (⩽10 d) compared to 6 months postpartum (Janzarik *et al* 2019). Our measurements were collected at rest without performing respiratory maneuvers and none of our patients experienced respiratory distress or received supplemental oxygen. As such, no substantial difference in PetCO2 between groups is expected. Future studies should investigate the impact of respiratory maneuvers (breath holding, hyperventilation) on cerebral autoregulation in postpartum patients with and without preeclampsia.

We also lacked a control group of young adults of either sex who were not pregnant or postpartum. Thus, we cannot make any inferences regarding the effects of the postpartum state on autoregulation. Prior studies have shown that cerebral autoregulation may be impaired even in healthy postpartum individuals (Janzarik *et al* 2019, Miller *et al* 2023). Additionally, patients with preeclampsia without severe features were not included in our study, so our findings cannot be extrapolated to all patients with preeclampsia. While one study found no differences in dynamic cerebral autoregulation in patients with preeclampsia with severe features compared to those without severe features, patients with preeclampsia with severe features trended towards worse autoregulatory function (Bergman *et al* 2021).

Our results may have been affected by the timing and administration of antihypertensive medications and intravenous magnesium sulfate. Over 90% of all preeclampsia participants received an antihypertensive or magnesium, or both, within 24 h prior to the study visit. Standard medications for the management of severe hypertension in pregnancy and postpartum include beta blockers (labetalol) and calcium channel blockers (nifedipine), both of which are vasoactive medications that may reduce CBF and improve cerebrovascular pulsatility and reactivity (Webb 2019). However, little is known about the effect of these antihypertensive medications on autoregulation in pregnancy and the postpartum period. Intravenous magnesium sulfate acts as a calcium channel agonist in vascular smooth muscle, causing vasodilation (Euser and Cipolla 2009) and may cause concentration-dependent vasodilation of the small distal cerebral vessels (Belfort and Moise 1992, Murata *et al* 2016). It is unclear how these physiological effects impacted autoregulation in our patient sample and how it may have consequently affected our curves. Additionally, the small number of patients who received magnesium within 24 h of the study may be a result of volunteer bias, where patients with active magnesium exposure are more acutely ill and less likely to consent to research studies.

Lastly, clinical characteristics differed significantly between groups, with higher proportions of obesity and preterm birth in the preeclampsia groups. There are limited studies regarding the effects of pregnancy on cerebral autoregulation even in healthy individuals, and even fewer data regarding the impact of risk factors such as chronic hypertension, obesity, preterm birth, and other adverse pregnancy outcomes on maternal cerebral autoregulation. Limited studies conflict regarding whether obesity is associated with impaired cerebral autoregulation pregnancy, with some investigators finding differences (Washio *et al* 2023) and others, no differences (van Veen *et al* 2015c). Regarding chronic hypertension and other hypertensive disorders in pregnancy, van Veen *et al* found that pregnant women with chronic hypertension or preeclampsia had impaired autoregulation compared to women with gestational hypertension and non-pregnant women (Van Veen *et al* 2015a). Maternal cerebral autoregulation in the early postpartum period is even less well studied and to our knowledge there are no studies regarding the effects of preterm birth on maternal autoregulation in the early postpartum period. These questions should all be investigated in future studies.

Given the limitations of this exploratory study, our results should be interpreted with caution and considered hypothesis-generating, to inform future research directions.

### 4.4. Conclusion

In this cohort of postpartum individuals, we produced aggregate static cerebral autoregulatory curves using data collected over 10 min. Future research should investigate methods for generating individual static autoregulatory curves using non-invasive techniques for short monitoring periods, potentially allowing clinicians to tailor blood pressure management for neuroprotection with bedside tools.

## Data Availability

The data cannot be made publicly available upon publication because they contain sensitive personal information. The data that support the findings of this study are available upon reasonable request from the authors.
